# Within-person associations of young adolescents’ physical activity across five primary locations: is there evidence of cross-location compensation?

**DOI:** 10.1186/s12966-017-0507-x

**Published:** 2017-04-20

**Authors:** Jordan A. Carlson, Tarrah B. Mitchell, Brian E. Saelens, Vincent S. Staggs, Jacqueline Kerr, Lawrence D. Frank, Jasper Schipperijn, Terry L. Conway, Karen Glanz, Jim E. Chapman, Kelli L. Cain, James F. Sallis

**Affiliations:** 1 Center for Children’s Healthy Lifestyles and Nutrition, Children’s Mercy Hospital, 610 E. 22nd St., Kansas City, MO 64108 USA; 2 University of Missouri Kansas City, Kansas City, MO USA; 3 University of Kansas, Lawrence, KS USA; 4 Seattle Children’s Research Institute and the University of Washington, Seattle, WA USA; 5 University of California San Diego, La Jolla, CA USA; 6 University of British Columbia, Vancouver, BC Canada; 7 University of Southern Denmark, Odense, Denmark; 8 University of Pennsylvania, Philadelphia, PA USA; 9 Urban Design 4 Health, Rochester, NY USA

**Keywords:** Built environment, Global Positioning Systems (GPS), Neighborhood, School

## Abstract

**Background:**

Youth are active in multiple locations, but it is unknown whether more physical activity in one location is associated with less in other locations. This cross-sectional study examines whether on days with more physical activity in a given location, relative to their typical activity in that location, youth had less activity in other locations (i.e., within-person associations/compensation).

**Methods:**

Participants were 528 adolescents, ages 12 to 16 (*M* = 14.12, *SD* = 1.44, 50% boys, 70% White non-Hispanic). Accelerometer and Global Positioning System devices were used to measure the proportion of time spent in moderate-to-vigorous physical activity (MVPA) in five locations: home, home neighborhood, school, school neighborhood, and other locations. Mixed-effects regression was used to examine within-person associations of MVPA across locations and moderators of these associations.

**Results:**

Two of ten within-participant associations tested indicated small amounts of compensation, and one association indicated generalization across locations. Higher at-school MVPA (relative to the participant’s average) was related to less at-home MVPA and other-location MVPA (Bs = −0.06 min/day). Higher home-neighborhood MVPA (relative to the participant’s average) was related to more at-home MVPA (B = 0.07 min/day). Some models showed that compensation was more likely (or generalization less likely) in boys and non-whites or Hispanic youth.

**Conclusions:**

Consistent evidence of compensation across locations was not observed. A small amount of compensation was observed for school physical activity, suggesting that adolescents partially compensated for high amounts of school activity by being less active in other locations. Conversely, home-neighborhood physical activity appeared to carry over into the home, indicating a generalization effect. Overall these findings suggest that increasing physical activity in one location is unlikely to result in meaningful decreases in other locations. Supporting physical activity across multiple locations is critical to increasing overall physical activity in youth.

**Electronic supplementary material:**

The online version of this article (doi:10.1186/s12966-017-0507-x) contains supplementary material, which is available to authorized users.

## Background

Despite the numerous benefits of physical activity in youth, current estimates indicate that most adolescents are not engaging in adequate amounts of physical activity. Data from 105 countries indicated that only 19% of adolescents worldwide achieved the recommended 60 min of physical activity per day based on self-report [1, 2], and there is evidence that this trend continues into adulthood [3]. Therefore, more research is needed to better understand patterns of physical activity in youth to inform efforts to promote physical activity.

Youth have the potential to be active in multiple locations, including within schools, homes, neighborhoods, and recreation areas such as parks, community centers, and sports facilities [4–9]. Because strategies for supporting physical activity differ by location, many public health intervention recommendations are location-specific (e.g., school-based physical activity, home-based screen time, neighborhood walking) [10–13]. Given the goal of increasing overall physical activity, it is important to understand whether increased physical activity in one location is related to decreased physical activity in other locations.

The compensation hypothesis [14] posits that youth maintain a presumed activity set-point by compensating for higher than usual activity at one time-point by engaging in lower than usual activity at a later time-point (i.e., compensation). Although several studies in youth did not find compensation to occur [15–17], there is some evidence suggesting that partial compensation occurs within and across days [18, 19]. There has been little to no examination of whether compensation occurs across *locations*, but this question is useful to investigate because it has implications for settings-based interventions. Such evidence would inform intervention strategies and priorities and help gauge the potential impact of setting-based interventions on overall physical activity. For example, if youth receive more physical activity at school but reduce their activity in other locations, coordinated multi-setting strategies may be needed to prevent such compensation from occurring.

The present study investigated how youth’s physical activity in one location was associated with physical activity in other locations. Specifically, the study examined whether an individual youth engaged in more or less activity in other locations when he/she was more active in a given location relative to his/her typical activity in that location (i.e., within-person associations). Moderators of compensation were also investigated to determine whether associations differed by participant characteristics (e.g., gender, age, neighborhood factors).

## Methods

### Participants and procedures

Data were from the Teen Environment and Neighborhood (TEAN) study of built environments and physical activity conducted in two US regions (Baltimore, MD/Washington, DC and Seattle/King County, WA) during 2009–2011. TEAN participants were 928 healthy adolescents ages 12–16 years, and one of their parents, selected from 447 census block groups representing high or low walkability and high or low income [20]. Data collection occurred during the school year and was balanced by season across the block group types. Overall participation rate (i.e., returned surveys divided by eligible contacts) was 36% and did not vary by neighborhood walkability or income. Comparisons of participants’ household demographics with census data indicated the study sample had higher education and household income compared to residents of the 447 census block groups in which participants lived. Regarding race/ethnicity, the study sample was comparable to census data for adolescent participants, with 34% being non-White or Hispanic versus 37% of adolescents in the census block groups from which participants were recruited. This study was approved by the sponsoring institution’s human subjects’ protection committee, parents provided informed consent, and adolescents provided assent.

Participants were asked to wear an accelerometer and Global Positioning System (GPS) tracker during all waking hours for 7 days, except during water activities. Present analyses included a subsample of 528 TEAN participants. Participants who were not given a GPS device (*N* = 130) and those who did not wear the provided accelerometer and GPS tracker together for ≥1 valid school day and ≥1 valid non-school day (*N* = 148) were excluded. Participants who attended homeschool, did not provide their school address, or had geocoding errors were excluded (*N* = 122). Participant demographic characteristics and MVPA did not differ significantly between the present subsample and the full sample.

### Measures

#### Demographic characteristics

Adolescents’ age, gender, and race/ethnicity (white non-Hispanic vs. non-white or Hispanic) were self-reported, and parents reported the highest level of education (college degree or higher vs. other) attained by any adult in the household.

#### Accelerometer-measured MVPA

Adolescents wore an Actigraph accelerometer on a belt at their left iliac crest during waking hours, with acceleration recorded at 30-s epochs. Multiple Actigraph models were used (7164, 85.2%; 71256, 5.1%; GT1M, 7.2%; GT3X, 2.5%), however, model type was not associated with MVPA in this study. MVPA was scored using the Evenson cut point for youth applied to the vertical axis acceleration counts [21], which has been shown to have excellent classification accuracy [22]. Groups of >60 sequential 30-s epochs (i.e., 30 min) with count = 0 were considered non-wear [23], and non-wear time was excluded from the data. Only days with ≥8 h of valid wear time were included.

#### GPS-derived variables

Participants wore a GlobalSat DG-100 GPS tracker, with latitude and longitude collected every 30 s when a GPS signal was attainable. Previous studies documented acceptable performance for tracking participants’ time and location patterns in epidemiological studies [24]. The Personal Activity and Location Measurement System (PALMS) [25] was used to merge GPS and accelerometer data and filter invalid GPS fixes caused by satellite interference; the devices were time-synchronized during initialization and linked in PALMS using their time stamp. Only days with ≥8 h of GPS signal during accelerometer wear time were included.

Home and school addresses were geocoded and incorporated into ArcGIS (ESRI, Inc; Redlands, CA) to create spatial buffers, and spatial analyses were performed in PostgreSQL to identify each participant’s amount of time and MVPA in 5 locations of interest. The locations were defined as follows: (1) home (50-m radius circular buffer around the point resulting from geocoding the home address), (2) home neighborhood (1-km street-network buffer around geocoded home address point, excluding the at-home circular buffer), (3) school (15-m buffer around geocoded school parcel), (4) school neighborhood (1-km street-network buffer around geocoded school point, excluding the at-school parcel buffer), and (5) all “other” locations (i.e., any location not included in the aforementioned 4 locations). Participants whose GPS indicated they never left their home over the monitoring period were considered to have not worn the device and were excluded. Participants who had overlap in their home neighborhood and school neighborhood buffers (20% of sample) were omitted from the analysis comparing home-neighborhood and school-neighborhoods MVPA. For all other models, overlapping time and MVPA were split evenly across the two overlapping buffers.

The resulting variables were computed at the day-level and were minutes per day of time present and MVPA occurring in each location. On a given day, if the participant spent 0 min in a location, MVPA for that location was scored as 0 min, with the exception of never leaving home during the monitoring period described above. Because school days and non-school days are distinct with regards to daily activities and patterns, a variable was derived to denote whether a day was a school or non-school day, with school day defined as any weekday the GPS showed the participant to be within the school parcel for ≥200 min.

### Analyses

Descriptive statistics were used to present MVPA and time across locations, separately for school days and non-school days. Next, day-level regression analyses were conducted using mixed effects regression to account for the nested data structure. Within-person associations were investigated between (a) MVPA in each location (independent variable) and overall MVPA (dependent variable), and (b) MVPA in each possible pair of locations. The five locations resulted in ten cross-location comparisons (each location was compared to the other four locations). Each participant’s location-specific MVPA values for school and non-school days were mean-centered on the participant’s average MVPA (across days) for that location on the given type of day (school or non-school) during the assessment period. This statistical approach for within-person associations is similar to creating differences scores (e.g., the participant’s MVPA in location A on a given day minus the participant’s average MVPA in location A across days), with the conceptual research question being “do days with above average MVPA in location A have below (i.e., compensation) or above (i.e., what we refer to as generalization) average MVPA in location B?” separately on school versus non-school days. All analyses were adjusted for participant mean-centered total time in each location.

Participant characteristics were not entered as covariates because between-person main effects were eliminated in mean-centering. The models tested *within*-person effects and any moderation of these effects by participant characteristics. Specifically, participant age, gender, race/ethnicity, and BMI, highest level of parent education, and neighborhood median income and high or low walkability [20] were tested as moderators of the within-person associations between MVPA in each pair of locations using interaction terms. Initially, each model also included a term to test the interaction between minutes of MVPA in the location (i.e., the independent variable) and school day (y/n) to explore differences across school days and non-school days. No school day interactions had a *p* value < .1, so the models reported combined school and non-school days. Unstandardized coefficients (B) for MVPA variables are presented and can be interpreted as the increase or decrease in the dependent variable (in minutes per day) associated with a 1-min increase in the independent variable. Interaction coefficients estimate the change in the MVPA effect (slope) associated with a 1-unit increase in the moderator.

## Results

Participants (*N* = 528) were from 317 block groups and 244 schools. Participants had a mean age of 14.12 (SD = 1.44), 50% were girls, 70% were White non-Hispanic, 46% lived in a high-walkability neighborhoods, 50% in high-income neighborhoods, and 52% resided in the Seattle/King County, WA region. Participants had a mean of 42.2 (SD = 22.5) minutes per day of overall MVPA across locations on school days, and 32.3 min per day on non-school days. Participants wore the accelerometer and GPS devices together for a mean of 3.9 (SD = 1.5) valid school days and 3.3 (SD = 1.7) valid non-school days, for a sample total of 3776 days. Location-specific MVPA is presented in Table 1.

**Table 1:** Young adolescents’ time and MVPA by location (*N* = 528 participants)

	Mean (SD) minutes/day of MVPA occurring in location		Mean (SD) proportion of overall wear time^a^ spent in location	
	School days	Non-school days	School days	Non-school days
At home	5.5 (6.7)	12.0 (14.2)	20.3% (15.5)	47.2% (34.8)
Home neighborhood	5.5 (9.3)	6.8 (11.6)	9.5% (12.6)	19.6% (28.2)
At school	23.2 (15.1)	0.6 (2.3)	57.7% (14.8)	1.2% (2.6)
School neighborhood	2.3 (4.3)	1.7 (5.0)	3.1% (5.1)	4.4% (12.8)
Other locations	5.6 (9.1)	10.9 (15.3)	9.3% (9.1)	27.3% (26.1)
All locations (Overall)	42.2 (22.5)	32.3 (21.8)	100%	100%

Note: Means and SDs were calculated across participants

^a^Refers only to the portion of the day the measurement devices were worn; Mean = 13.3, SD = 1.8 h per day

For each location, days when adolescents had more MVPA in the location as compared to his/her average in that location had more overall MVPA (i.e., a 1-min/day increase in location-specific MVPA was related to a 0.88–1.03 min/day increase in overall MVPA; Table 2). A regression coefficient <1.0 indicated that some compensation had occurred, whereas a regression coefficient >1.0 indicated that generalization across locations had occurred. The largest compensation effect was observed for at-school MVPA (B = 0.88; i.e., 12% compensation). The coefficients for home-neighborhood and school-neighborhood MVPA indicated that each minute of home-neighborhood MVPA was associated with 1.03 min/day of overall MVPA, and each minute/day of school-neighborhood MVPA was associated with 1.01 min/day of overall MVPA (i.e., 1–3% generalization).

**Table 2:** Within-person associations (i.e., compensation) between MVPA in each location and overall MVPA (*N* = 3776 days)

	Associations with overall MVPA	
	B (SE) minutes/day	*p*
At home MVPA	0.95 (0.03)	<.001
Home neighborhood MVPA	1.03 (0.04)	<.001
At school MVPA	0.88 (0.03)	<.001
School neighborhood MVPA	1.01 (0.09)	<.001
Other locations MVPA	0.90 (0.03)	<.001

Note: All models were adjusted for participant age, gender, race/ethnicity, and BMI, parent education, neighborhood income and walkability, time in location, and average (across days of monitoring) time and MVPA in location

Two of the ten cross-location comparisons indicated compensation, and one of the 10 indicated generalization (Table 3). On days when adolescents had more at-school MVPA relative to their average at-school MVPA, they had less at-home MVPA and other-location MVPA (both Bs = -0.06 min/day, i.e., each 6% compensation). On days when adolescents had more home-neighborhood MVPA relative to their average, they had more at-home MVPA (B = 0.07 min/day; i.e., 7% generalization).

**Table 3:** Within-person associations (i.e., compensation) among MVPA minutes/day across 5 primary locations (*N* = 3776 days)

	Compensation effect		Factors *p* < .05 associated with more compensation or less generalization (interaction B)^a^
	B (95% CI) minutes/day	*p*	
Associations across locations			
Home neighborhood → At home	0.07 (0.04, 0.10)	<.001	High income (-0.09) Non-White or Hispanic (-0.10) +1 year in age (-0.03)
Home neighborhood → School neighborhood^b^	0.00 (−0.01, 0.02)	.806	[none]
Home neighborhood → Other locations	0.01 (−0.04, 0.06)	.627	Boys (−0.14)
At school → At home	−0.06 (−0.09, −0.03)	<.001	Boys (−0.07) Non-White or Hispanic (-0.11)
At school → Home neighborhood	−0.02 (−0.05, 0.01)	.170	[none]
At school → School neighborhood	0.01 (−0.01, 0.02)	.483	[none]
At school → Other locations	−0.06 (−0.11, −0.02)	.006	[none]
At home → School neighborhood	−0.02 (−0.04, 0.00)	.106	[none]
Other locations → School neighborhood	0.00 (−0.01, 0.01)	.861	[none]
Other locations → At home	0.00 (−0.04, 0.03)	.762	Boys (−0.06) Higher walkability (−0.10)

Note: The independent variable appears before the arrow and the dependent variable appears after the arrow. Daily MVPA in each location was participant mean centered so that the effects would reflect within person differences. All models were adjusted for daily time in location which was also participant mean centered

^a^Moderators tested were participant gender, age, race/ethnicity, and BMI percentile, neighborhood walkability and income, and parent education

^b^Excluded participants with overlap between their home and school neighborhood (20% of sample)

**Fig. 1:**
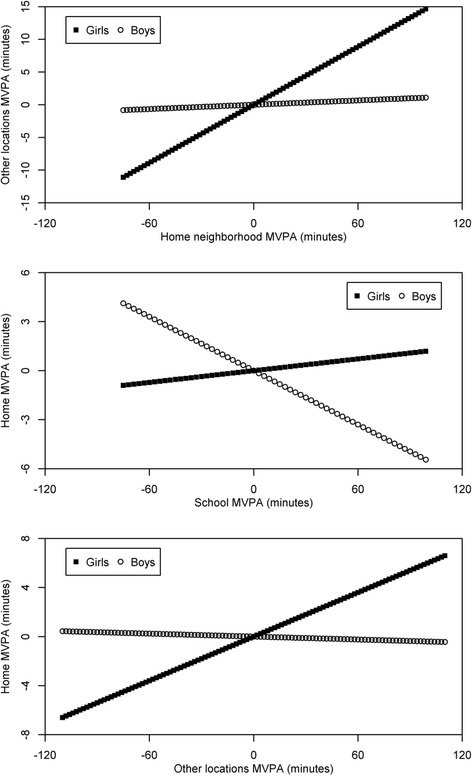
Gender differences in within-person associations among MVPA minutes/day across locations

Findings regarding whether participant factors moderated associations of MVPA between each pair of locations are summarized in Table 3 and presented in full detail in the Additional file 1: Table S1. Gender was a significant moderator in three of the 10 models, with girls showing generalization and boys no effect in two models, and boys showing compensation and girls no effect in one model (see Fig. 1). Race/ethnicity was a significant moderator in two models, with non-whites or Hispanics compensating more than white non-Hispanics. Neighborhood income, child age, and neighborhood walkability each emerged as moderators in only one of the 10 models.

## Discussion

The present study found limited consistent evidence of compensation or generalization of adolescent physical activity across locations. Thus, amounts of physical activity in multiple locations were mainly independent from each other. Small compensation effects were observed for physical activity between the school location and at-home/other locations (12% total), whereas a small generalization effect was observed between the home neighborhood and at-home location (7%). Results indicate the home neighborhood is a promising location for interventions targeting physical activity in adolescents, without concern of compensation. Programs targeting school physical activity may not result in a 1:1 contribution to overall physical activity (i.e., each minute of school physical activity may not equate to a full minute of overall physical activity because of potential compensation in other locations) but still provide meaningful contributions.

There was no consistent evidence that compensation across locations was moderated by participant age (although the age range was narrow), participant BMI, neighborhood walkability, neighborhood income, or parent education. There was some evidence (3 of 10 interactions tested) of moderation by gender, with boys being more likely to compensate than girls in one of the location comparisons and less likely to show generalization in two of the location comparisons. There was some evidence (2 of 10 interactions tested) that adolescents who identified as white non-Hispanic compensated less (or showed more generalization) than their counterparts. Two of the three gender interactions, and both of the race/ethnicity interactions included at-home physical activity. It could be that home physical activity is less structured and less social than physical activity that occurs elsewhere, making it and its propensity for being compensated for more likely to be influenced by individual-level factors. Although amount of physical activity in each location differed across school and non-school days (presented in more detail in a previous publication) [9], compensation across locations did not differ between school and non-school days. This suggests that whether a child attends school or not on a given day does not appear to be relevant to concerns about compensation. This was somewhat surprising given that time in school on school days is considerable and could conceivably make compensation across non-school locations more likely.

Most studies that have investigated compensation have done so across time, especially days, rather than across locations [15–19]. These studies have had mixed results, but some have found evidence of compensation [16, 18, 19, 26]. One study found that children partially compensated within days (across time periods) and between days by engaging in fewer steps directly following a day (or time period) with more than average steps [19]. Comparability of previous findings on compensation is challenging because unlike the present study, prior studies did not account for natural variations in time use by adjusting for the total amount of time participants spent in specific locations. Present findings provided limited evidence of compensation or generalization, with the only evidence of compensation effects being specific to the school location. Thus, these effects are unlikely to have major impacts on youth’s overall physical activity. This finding suggests that, although each minute of school-based physical activity may not translate to a full additional minute of overall physical activity, increasing physical activity opportunities in school is an important strategy for improving overall physical activity, which is in agreement with previous studies and national recommendations [12, 27, 28]. To address the potential for compensation, parents should encourage and support their child to be physically active outside of school on all days, including days with Physical Education (PE) or school sports.

Interestingly, generalization of physical activity across locations occurred between the home neighborhood and at-home location. This association could have been due to activities that took place in and/or directly outside of the home and carried over into the neighborhood (i.e., crossed over the home buffer into the neighborhood buffer), such as playing outdoors and active travel. Given that home-neighborhood physical activity showed a generalization effect and that youth are more likely to be physically active when in their neighborhood than when at home, at school, and in other locations [9], supporting neighborhood-based physical activity appears to be a particularly promising strategy for increasing overall physical activity in youth. Strategies that increase neighborhood-based physical activity include outdoor walking, neighborhood play and “Play Streets,” [29], and active travel to school. Review papers have shown that active travel to school does not lead to compensation in physical activity [30, 31]. Consistent evidence is accumulating on the importance of supporting active travel in youth [32–35], and Safe Routes to School programs were effective in several recent evaluations [36–39]. More widespread implementation of Safe Routes to School programs, as well as other evidence-based efforts, are needed to increase the currently low rates of neighborhood physical activity and active travel in youth as shown by the present study [40] and others [30, 40–42]. Effective strategies are likely multilevel and include combinations of built environment, neighborhood safety, and social/interpersonal strategies [43]. The present finding that physical activity may generalize across locations supports comprehensive systems approaches such as those that target multiple strategies in multiple locations [44, 45].

The compensation effects that were found were specific to school-based activity, which has been observed in previous studies [26], whereas the generalization effect was specific to home-neighborhood physical activity. It is possible that compensation is more likely to occur when the physical activity is organized, structured, or even mandated (e.g., physical education class) than when physical activity is discretionary, because discretionary physical activity may be less likely to be perceived as “exercise”. For example, neighborhood activity is most likely to consist of active play and/or active transportation, activities performed for fun or out of necessity to accomplish another objective (e.g., getting to school or other destinations) and less likely to be viewed as “exercise”. School-based activity could include sports, which are likely perceived as highly active. Adolescents may engage in lower activity levels outside of sports to retain energy for sports, or be inactive after participating in sports because of fatigue. Parents or children may think that if the child participated in sports or PE then he/she does not need to be active at home. These beliefs can be detrimental because, based on the present and other findings, adolescents accrue less than half of the recommended 60 min per day of physical activity at school.

### Strengths, limitations, and research gaps

The present study utilized a large sample of adolescents in two US regions, within-person analyses, and location-specific estimates of objectively-assessed physical activity derived from GPS and accelerometers, which were methodological strengths. Going beyond main effects, the present study used interaction tests, which revealed no differences between school days and non-school days, with gender as the only consistent moderator of compensation effects across locations. These strengths enhance confidence in the novel contribution of examining the possibility of cross-location compensation. For limitations, the use of 30-s epochs with accelerometers could have underestimated participants’ physical activity, as some authors recommend shorter epochs [22, 46]. Another limitation was the potential for misclassification from the GPS, such as when signals were unreliable in some indoor environments, and the observational nature of the study, which limited understanding of causality. Since “other locations” were simply any location outside of the home and school neighborhoods, future studies could provide more specificity by documenting physical activity in key types/categories of locations, such as recreation locations. Sports facilities, parks, and community centers are known to be important locations for physical activity and should be investigated in future studies [47]. Another limitation was that indoor physical activity may have been underestimated because we omitted time with missing GPS information (e.g., due to signal loss) from the present analyses. Methods have recently been validated for imputing missing geocoordinate information and should be considered in future studies [48].

Overall, limited evidence of compensation or generalization across locations was found, but future studies could investigate whether compensation differs by activity type (e.g., sports vs. other school-based activity), which was not feasible in the present study. Future studies should also investigate whether youth compensate for high amounts of physical activity in one location by having more than typical sedentary time in other locations, which is plausible and has important health implications. The present study did not investigate compensation by time (e.g., within or across days), which has been observed in some studies [18, 19], but findings have been inconsistent [15–17].

## Conclusions

The primary finding was that adolescent physical activity in one location was mainly independent of activity in other locations. Thus, promoting physical activity in all locations could be expected to contribute to increased activity levels. However, small amounts of both compensation and generalization across locations were observed. Compensation was observed between school and at-home/other locations, and boys, and non-whites or Hispanics tended to be more likely to compensate or less likely to show generalization across locations. The generalization effect was specific to neighborhood-based physical activity. Overall, these findings indicated that increasing physical activity in one location is not likely to result in meaningful decreases in other locations. Supporting physical activity across multiple locations is critical to increasing overall physical activity in youth. Targeting neighborhood-based activity should be a high priority, given its critical role in overall levels of physical activity.

## Additional file

Additional file 1: Table S1. Interactions of within-person associations (i.e., compensation) among MVPA minutes/day across 5 primary locations (N = 3776 days). (DOC 41 kb)

## Abbreviations

GPS: Global positioning system
MVPA: Moderate-to-vigorous physical activity
PALMS: Personal activity and location measurement system
TEAN: Teen environment and neighborhood

## References

[1] Hallal PC, Andersen LB, Bull FC, Guthold R, Haskell W, Ekelund U (2012). Global physical activity levels: surveillance progress, pitfalls, and prospects. Lancet.

[2] Sallis JF, Bull F, Guthold R, Heath GW, Inoue S, Kelly P, Oyeyemi AL, Perez LG, Richards J, Hallal PC (2016). Progress in physical activity over the Olympic quadrennium. Lancet.

[3] Telama R (2009). Tracking of physical activity from childhood to adulthood: a review. Obes Facts.

[4] Grow HM, Saelens BE, Kerr J, Durant NH, Norman GJ, Sallis JF (2008). Where are youth active? Roles of proximity, active transport, and built environment. Med Sci Sports Exerc.

[5] Kneeshaw-Price S, Saelens BE, Sallis JF, Glanz K, Frank LD, Kerr J, Hannon PA, Grembowski DE, Chan KC, Cain KL (2013). Children’s objective physical activity by location: why the neighborhood matters. Pediatr Exerc Sci.

[6] Rainham DG, Bates CJ, Blanchard CM, Dummer TJ, Kirk SF, Shearer CL (2012). Spatial classification of youth physical activity patterns. Am J Prev Med.

[7] Jones AP, Coombes EG, Griffin SJ, van Sluijs EM (2009). Environmental supportiveness for physical activity in English schoolchildren: a study using Global Positioning Systems. Int J Behav Nutr Phys Act.

[8] Klinker CD, Schipperijn J, Christian H, Kerr J, Ersboll AK, Troelsen J (2014). Using accelerometers and global positioning system devices to assess gender and age differences in children’s school, transport, leisure and home based physical activity. Int J Behav Nutr Phys Act.

[9] Carlson JA, Schipperijn J, Kerr J, Saelens BE, Natarajan L, Frank LD, Glanz K, Conway TL, Chapman JE, Cain KL, Sallis JF (2016). Locations of physical activity as assessed by GPS in young adolescents. Pediatrics.

[10] U.S. Department of Health and Human Services. 2008 physical activity guidelines for Americans. Available at: http://www.health.gov/paguidelines/pdf/paguide.pdf. Accessed 3 Feb 2017.

[11] Healthy People 2020. [http://www.healthypeople.gov/2020/topicsobjectives2020/]. Accessed 3 Feb 2017.

[12] Institute of Medicine (2013). Educating the student body: taking physical activity and physical education to school.

[13] Pate RR, Davis MG, Robinson TN, Stone EJ, McKenzie TL, Young JC (2006). Promoting physical activity in children and youth: a leadership role for schools: a scientific statement from the American Heart Association Council on Nutrition, Physical Activity, and Metabolism (Physical Activity Committee) in collaboration with the Councils on Cardiovascular Disease in the Young and Cardiovascular Nursing. Circulation.

[14] Rowland TW (1998). The biological basis of physical activity. Med Sci Sports Exerc.

[15] Goodman A, Mackett RL, Paskins J (2011). Activity compensation and activity synergy in British 8–13 year olds. Prev Med.

[16] Gomersall SR, Rowlands AV, English C, Maher C, Olds TS (2013). The ActivityStat hypothesis: the concept, the evidence and the methodologies. Sports Med.

[17] Baggett CD, Stevens J, Catellier DJ, Evenson KR, McMurray RG, He K, Treuth MS (2010). Compensation or displacement of physical activity in middle-school girls: the Trial of Activity for Adolescent Girls. Int J Obes (Lond).

[18] Ridgers ND, Timperio A, Cerin E, Salmon J (2014). Compensation of physical activity and sedentary time in primary school children. Med Sci Sports Exerc.

[19] Ridgers ND, Timperio A, Cerin E, Salmon J (2015). Within- and between-day associations between children’s sitting and physical activity time. BMC Public Health.

[20] Frank LD, Sallis JF, Saelens BE, Leary L, Cain K, Conway TL, Hess PM (2010). The development of a walkability index: application to the Neighborhood Quality of Life Study. Br J Sports Med.

[21] Evenson KR, Catellier DJ, Gill K, Ondrak KS, McMurray RG (2008). Calibration of two objective measures of physical activity for children. J Sports Sci.

[22] Trost SG, Loprinzi PD, Moore R, Pfeiffer KA (2011). Comparison of accelerometer cut points for predicting activity intensity in youth. Med Sci Sports Exerc.

[23] Cain KL, Sallis JF, Conway TL, Van Dyck D, Calhoon L (2013). Using accelerometers in youth physical activity studies: a review of methods. J Phys Act Health.

[24] Wu J, Jiang C, Liu Z, Houston D, Jaimes G, McConnell R (2010). Performances of different global positioning system devices for time-location tracking in air pollution epidemiological studies. Environ Health Insights.

[25] Physical Activity Location Measurement System. [http://cwphs.ucsd.edu/palms]. Accessed 3 Feb 2017.

[26] Fremeaux AE, Mallam KM, Metcalf BS, Hosking J, Voss LD, Wilkin TJ (2011). The impact of school-time activity on total physical activity: the activitystat hypothesis (EarlyBird 46). Int J Obes (Lond).

[27] Centers for Disease Control and Prevention (2013). Comprehensive school physical activity programs: a guide for schools.

[28] American Alliance for Health PE, Recreation and Dance. Comprehensive school physical activity programs: helping students achieve 60 minutes of physical activity each day. Reston: 2013.

[29] D’Haese S, Van Dyck D, De Bourdeaudhuij I, Deforche B, Cardon G (2015). Organizing “Play Streets” during school vacations can increase physical activity and decrease sedentary time in children. Int J Behav Nutr Phys Act.

[30] Faulkner GEJ, Buliung RN, Flora PK, Fusco C. Active school transport, physical activity levels and body weight of children and youth: a systematic review. Prev Med. 2009;48. 10.1016/j.ypmed.2008.10.017 19014963

[31] Larouche R, Saunders TJ, Faulkner G, Colley R, Tremblay M (2014). Associations between active school transport and physical activity, body composition, and cardiovascular fitness: a systematic review of 68 studies. J Phys Act Health.

[32] Lubans DR, Boreham CA, Kelly P, Foster CE (2011). The relationship between active travel to school and health-related fitness in children and adolescents: a systematic review. Int J Behav Nutr Phys Act.

[33] Cooper AR, Page AS, Foster LJ, Qahwaji D (2003). Commuting to school: are children who walk more physically active?. Am J Prev Med.

[34] Cooper AR, Andersen LB, Wedderkopp N, Page AS, Froberg K (2005). Physical activity levels of children who walk, cycle, or are driven to school. Am J Prev Med.

[35] Berrigan D, Troiano RP, McNeel T, DiSogra C, Ballard-Barbash R (2006). Active transportation increases adherence to activity recommendations. Am J Prev Med.

[36] McDonald NC, Steiner RL, Lee C, Rhoulac Smith T, Zhu X, Yang Y (2014). Impact of the safe routes to school program on walking and bicycling. J Am Plann Assoc.

[37] Muennig PA, Epstein M, Li G, DiMaggio C (2014). The cost-effectiveness of New York City’s safe routes to school program. Am J Public Health.

[38] Stewart O, Moudon AV, Claybrooke C (2014). Multistate evaluation of safe routes to school programs. Am J Health Promot.

[39] Impact of Safe Routes to School Programs on Walking and Biking. [http://activelivingresearch.org/SRTSreview]. Accessed 3 Feb 2017.

[40] Carlson JA, Saelens BE, Kerr J, Schipperijn J, Conway TL, Frank LD, Chapman JE, Glanz K, Cain KL, Sallis JF (2015). Association between neighborhood walkability and GPS-measured walking, bicycling and vehicle time in adolescents. Health Place.

[41] McDonald NC (2007). Active transportation to school: trends among U.S. schoolchildren, 1969–2001. Am J Prev Med.

[42] Ham SA, Macera CA, Lindley C (2005). Trends in walking for transportation in the United States, 1995 and 2001. Prev Chronic Dis.

[43] Sallis JF, Owen N, Fisher E, Glanz K, Rimer BK, Viswanath K (2015). Ecological models of health behavior. Health behavior: theory, research, and practice.

[44] President’s Council on Fitness Sports and Nutrition (2013). Physical activity guidelines for Americans mid-course report: strategies to increase physical activity among youth.

[45] Institute of Medicine (2012). Accelerating progress in obesity prevention: solving the weight of the nation.

[46] Baquet G, Stratton G, Van Praagh E, Berthoin S (2007). Improving physical activity assessment in prepubertal children with high-frequency accelerometry monitoring: a methodological issue. Prev Med.

[47] Klinker C, Schipperijn J, Kerr J, Ersbøll A, Troelsen J (2014). Context-specific outdoor time and physical activity among school-children across gender and age: using accelerometers and GPS to advance methods. Front Public Health.

[48] Meseck K, Jankowska MM, Schipperijn J, Natarajan L, Godbole S, Carlson J, Takemoto M, Crist K, Kerr J (2016). Is missing geographic positioning system data in accelerometry studies a problem, and is imputation the solution?. Geospat Health.

