# Supplementary material for: Within-person associations of young adolescents’ physical activity across five primary locations: is there evidence of cross-location compensation?
Source: Int J Behav Nutr Phys Act. 2017 Apr 20;14:50. doi: 10.1186/s12966-017-0507-x (PMC5397771; doi:10.1186/s12966-017-0507-x)
Supplement: Additional file 1: Table S1. — Interactions of within-person associations (i.e., compensation) among MVPA minutes/day across 5 primary locations (N = 3776 days). (DOC 41 kb) [file 12966_2017_507_MOESM1_ESM.doc]

**Additional file 1: Table S1. Interactions of within-person associations (i.e., compensation) among MVPA minutes/day across 5 primary locations (N = 3776 days)**

|  | **Home neighborhood → At home** | **Home neighborhood → School neighborhood^a^** | **Home neighborhood → Other locations** | **At school → At home** | **At school → Home neighborhood** | **At school → School neighborhood** | **At school → Other locations** | **At home → School neighborhood** | **Other locations → School neighborhood** | **Other locations → At home** |
| --- | --- | --- | --- | --- | --- | --- | --- | --- | --- | --- |
|  | **B (95% CI)** | **B (95% CI)** | **B (95% CI)** | **B (95% CI)** | **B (95% CI)** | **B (95% CI)** | **B (95% CI)** | **B (95% CI)** | **B (95% CI)** | **B (95% CI)** |
| **Boys vs. girls** | 0.02 (-0.06, 0.08) | 0.01 (-0.01, 0.04) | 0.14 (0.04, 0.24) | 0.07 (0.01, 0.12) | 0.02 (-0.01, 0.06) | 0.02 (0, 0.04) | 0.05 (-0.01, 0.11) | 0.02 (-0.01, 0.05) | 0.01 (-0.02, 0.03) | 0.06 (0.01, 0.12) |
| **High vs. low walkability** | -0.06 (-0.14, 0.01) | -0.02 (-0.04, 0) | 0.05 (-0.06, 0.16) | 0.01 (-0.05, 0.07) | 0.03 (-0.01, 0.07) | 0.01 (-0.02, 0.03) | 0 (-0.07, 0.06) | 0.01 (-0.02, 0.04) | 0 (-0.02, 0.02) | -0.1 (-0.15, -0.04) |
| **High vs. low income** | -0.09 (-0.16, -0.03) | 0 (-0.02, 0.02) | -0.06 (-0.15, 0.03) | 0.02 (-0.05, 0.09) | -0.02 (-0.06, 0.02) | -0.01 (-0.03, 0.01) | -0.03 (-0.1, 0.02) | 0 (-0.03, 0.03) | 0 (-0.02, 0.02) | 0.02 (-0.03, 0.07) |
| **White non-Hispanic vs. other** | 0.1 (0.03, 0.18) | -0.01 (-0.03, 0.01) | -0.05 (-0.15, 0.06) | 0.11 (0.03, 0.19) | 0.02 (-0.03, 0.06) | 0 (-0.03, 0.03) | 0.06 (-0.02, 0.15) | 0.02 (-0.01, 0.06) | 0.01 (-0.02, 0.03) | -0.02 (-0.07, 0.04) |
| **College degree y vs. no** | 0.05 (-0.02, 0.14) | 0 (-0.01, 0.02) | 0.02 (-0.08, 0.12) | -0.02 (-0.08, 0.05) | 0.02 (-0.04, 0.08) | 0.01 (-0.01, 0.03) | -0.02 (-0.09, 0.04) | 0.01 (-0.03, 0.04) | 0 (-0.02, 0.02) | -0.02 (-0.08, 0.03) |
| **Age** | -0.03 (-0.06, -0.01) | 0 (-0.01, 0) | 0.03 (0, 0.06) | 0.01 (-0.02, 0.03) | 0 (-0.01, 0.02) | 0 (-0.01, 0) | 0.02 (0, 0.04) | 0 (-0.01, 0.01) | 0 (-0.01, 0.01) | 0.01 (0, 0.03) |
| **BMI percentile^b^** | -0.04 (-0.08, 0) | 0 (-0.01, 0.01) | 0.04 (-0.01, 0.1) | 0.03 (0, 0.07) | 0 (-0.02, 0.02) | 0.01 (0, 0.02) | -0.02 (-0.06, 0) | -0.01 (-0.03, 0) | 0.01 (-0.01, 0.02) | 0.01 (-0.02, 0.04) |
| **Note: The independent variable appears before the arrow and the dependent variable appears after the arrow. Reference categories for the dichotomous independent variables appear after the “vs.”. Daily MVPA in each location was participant mean centered so that the effects would reflect within person differences. All models were adjusted for daily time in location which was also participant mean centered.**  **^a^Excluded participants with overlap between their home and school neighborhood (20% of sample)**  **^b^Scaled by dividing percentile by 25 (roughly 1 standard deviation).** | | | | | | | | | | |
